# Detection of Monkeypox Virus Clade Ib DNA in Wastewater Solids at Wastewater Treatment Plants, United States

**DOI:** 10.3201/eid3110.250270

**Published:** 2025-10

**Authors:** Alexandria B. Boehm, Marlene K. Wolfe, Amanda L. Bidwell, Bradley J. White, Bridgette Shelden, Dorothea Duong

**Affiliations:** Author affiliations: Stanford University, Stanford, California, USA (A.B. Boehm, A.L. Bidwell); Emory University, Atlanta, Georgia, USA (M.K. Wolfe); Google LLC, Mountain View, California, USA (B.J. White); Verily Life Sciences LLC, South San Francisco, California, USA (B.J. White, B. Shelden, D. Duong)

**Keywords:** monkeypox virus, MPXV, clade Ib, mpox, viruses, wastewater, United States

## Abstract

We used a sensitive and specific PCR to detect monkeypox virus clade Ib DNA from >3,000 wastewater samples collected prospectively across the United States. Monkeypox virus clade Ib DNA was detected in 1 sample from a location with a confirmed case; it was not detected in locations with no confirmed cases.

Monkeypox virus (MPXV), which causes mpox, is divided into 2 clades, I and II (*1*). MPXV clade I (now Ia) is endemic in Central Africa and causes mpox outbreaks primarily in children, (*1*) but in 2024, a new subclade, clade Ib, was identified in the Democratic Republic of the Congo (*1*). Clade Ib has spread rapidly since its emergence, primarily among adults through sexual contact (*1*). As of January 1, 2024, nearly 36,000 mpox clade Ib cases and >100 deaths have been confirmed in Central and Eastern Africa (*2*). Since February 12, 2025, at least 4 travel-associated mpox clade Ib cases have been confirmed in the United States, 1 each in California (*3*), Georgia (*2*), New Hampshire (*4*), and New York (*5*). Outbreaks of clade I mpox have previously been associated with a higher case-fatality rate than for clade II, but data to establish the severity of clade Ib during the ongoing outbreak are limited (*1*).

MPXV clade II DNA can be detected in wastewater solids, and its concentrations correlate to community incident cases of infections (*6*). Because MPXV clade II DNA is shed in saliva, feces, urine, and semen and from skin lesions of infected persons (*7*), it might end up in wastewater (*8*). 

We have been prospectively measuring MPXV clade II DNA across the United States for use by public health organizations to identify outbreaks (*9*). Recent work has established that MPXV clade I DNA is shed via excretions that enter wastewater (*10*). We aimed to test if MPXV clade Ib DNA can be detected in wastewater solids to provide information on potential spread. 

## The Study

We used a previously published and validated MPXV clade Ib hydrolysis-probe PCR (*11*) (Table) in a droplet digital PCR format. We confirmed the specificity and sensitivity of the assay in vitro and in silico (Appendix).

**Table:** Monkeypox virus clade Ib probe and primers used to detect mpox in wastewater solids at wastewater treatment plants, United States

Probe or primer	Sequence, 5′→3′
Probe	ATATTCAGGCGCATATCCACCCACGT
Forward primer	AAGACTTCCAAACTTAATCACTCCT
Reverse primer	CGTTTGATATAGGATGTGGACATTT

We retrospectively applied the assay to 10 wastewater samples (Appendix). The 10 samples were collected from a wastewater treatment plant (WWTP) serving ≈750,000 people in San Francisco, California, USA. MPXV clade II DNA was previously detected at both high and low concentrations in those samples, and the samples were collected at a time when MPXV clade Ib was not known to be circulating in the United States (Appendix). Samples were stored at –80°C as purified nucleic acids for 1–3 years before analysis. We obtained the nucleic acids by using previously published methods (*9*) (Appendix). We confirmed limited degradation of nucleic acids during storage by also measuring SARS-CoV-2 RNA concentration and comparing the measurements to those obtained from the same samples before storage (Appendix). MPXV clade Ib DNA concentrations were nondetectable in the 10 samples, supporting the specificity of the molecular assay.

We began prospective measurements of MPXV clade Ib DNA in samples from 147 WWTPs located in 40 states (Figure). Samples were collected during November 22, 2025–January 31, 2025, typically 3 times/week. Full descriptions of WWTP locations and the number of samples and the time during which they were collected are provided (Appendix). Per WWTP, 11–71 samples were tested (median 21 samples/WWTP).

**Figure:**
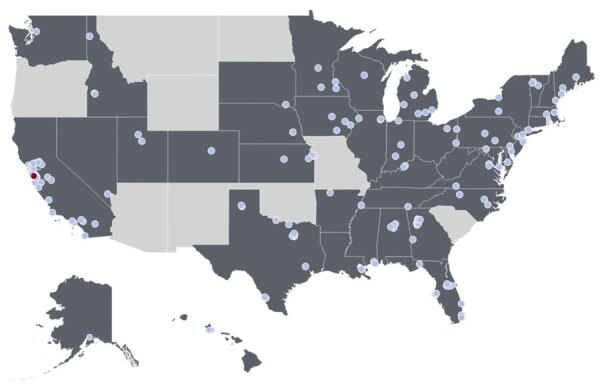
Locations of wastewater treatment plants (WWTPs) enrolled in study to detect monkeypox virus (MPXV) clade Ib in wastewater solids at WWTPs, United States. Dark gray shading indicates states with enrolled sites. The red dot represents the location of the WWTP where there was a positive detection of MPXV clade Ib (n = 1), and the light blue dots represent participating WWTPs where no samples were positive for MPXV (n = 146).

We conducted sampling as previously described (*9*) (Appendix). After collection, we stored samples at 4°C, shipped them to the laboratory on ice, and processed to completion within 48 hours of receipt at the laboratory. We do not expect the time between sample collection and analysis to affect target quantification because our work and previously published studies (*12*–*14*) show limited decay of the short length nucleic-acid targets over multiple weeks at 4°C. We collected and analyzed a total of 3,292 samples.

We processed samples and extracted and purified their nucleic acids from wastewater solids using previously described methods (*9*) (Appendix). We measured concentrations of MPXV clade Ib DNA by using droplet digital PCR on 6–10 replicates for each sample. We ran extraction and PCR positive and negative controls on each 96-well plate. We merged results from replicate wells for analysis. Concentrations are presented as copies per gram of dry weight. For a sample to be scored as a positive, there had to be >3 positive droplets. The lowest measurable concentration was ≈1,000 copies/g (3 positive droplets). We reported errors as SDs of the measurements obtained from QX Manager software version 2.0 (Bio-Rad Laboratories, https://www.bio-rad.com). Data are available through the Stanford Digital Repository (https://doi.org/10.25740/vk458br0887).

Only 1 sample of 3,292 tested positive. In the positive sample, the concentration of MPXV clade Ib DNA was 1,174 copies/g (68% CI 674–1,875 copies/g; 4 positive droplets). The positive sample was obtained from a sewershed in California, USA, serving ≈199,000 persons, including a person with confirmed clade Ib mpox (*3*). The positive sample was collected on December 16, 2024, twenty-nine days after a news story broke of a travel-associated infection in the community. Sewer transit times are <1 day. MPXV clade I DNA shedding from infected patients via skin, feces, saliva, and mucus can persist for >20 days after symptom onset (*10*). It is therefore possible that DNA from excretions of the infected person led to the positive detection.

A total of 3,291 samples were negative for MPXV clade Ib DNA, consistent with a lack of known cases across the United States. A negative sample is defined as a sample with <3 positive droplets across all generated droplets for that sample. Negative controls and positive controls in this study performed as expected (Appendix). There have also been confirmed cases of MPXV clade Ib infections in Georgia, New Hampshire, and New York. However, according to news reports, those persons were not expected to be within a WWTP sewershed included in this study, and the New Hampshire and New York cases were first reported after the last sample in this study was collected.

## Conclusions

In this study, only 1 wastewater sample was positive for MPXV clade Ib DNA, and that sample was from a location where there was a known, travel-associated case. Samples from the same location before and after the positive sample were negative, and no secondary cases were reported. Variability in concentrations of rare nucleic acid targets in wastewater should be expected and is caused by many factors. Wastewater MPXV DNA concentrations might vary temporally because of variability in shedding by infected persons, movement of people into and out of the sewersheds, or deliveries of septic wastes to the system. In addition, spatial heterogeneities in MPXV DNA within the solid matrix might exist. To address variability, we processed high frequency samples and ran 6–10 replicates and numerous controls to ensure high quality measurements.

This study establishes methods for measuring MPXV clade Ib DNA in wastewater solids for surveillance of MPXV clade Ib infections. Results from our analysis of wastewater solids samples are consistent with information on the presence and absence of MPXV clade Ib infections in the included WWTP sewersheds during the study, and the results suggest wastewater monitoring for MPXV clade Ib might be useful to monitor disease outbreaks. The methods used to measure MPXV clade Ib could be used to monitor MPXV clade Ia as well. 

Appendix Additional information about detection of monkeypox virus clade Ib in wastewater solids at wastewater treatment plants, United States.
