# Supplementary material for: Detection of Monkeypox Virus Clade Ib DNA in Wastewater Solids at Wastewater Treatment Plants, United States
Source: Emerg Infect Dis. 2025 Oct;31(10):1984–7. doi: 10.3201/eid3110.250270 (PMC12483025; doi:10.3201/eid3110.250270)
Supplement: Appendix — Additional information about detection of monkeypox virus clade Ib in wastewater solids at wastewater treatment plants, United States. [file 25-0270-Techapp-s1.pdf]

Article DOI: https://doi.org/10.3201/eid3110.250270

EID cannot ensure accessibility for supplementary materials supplied by authors. Readers who have difficulty accessing supplementary content should contact the authors for assistance.

Detection of Monkeypox Virus Clade Ib in Wastewater Solids at Wastewater Treatment Plants, United States

Appendix

Methods

Assay choice

A previously published assay (*1*) was used to detect clade Ib mpox virus genomic DNA in wastewater (Table, main text). This assay was designed in response to the emergence of clade Ib, including the introduction of a deletion in the complement binding protein (C3L) gene which has been commonly used to identify clade I mpox viruses. The assay spans the deletion; targeting regions upstream and downstream of the C3L deletion in Clade Ib. The original authors validated the assay by testing clinical samples from the clade Ib outbreak in the Democratic Republic of Congo (*1*).

In silico assay testing

We downloaded mpox genome sequences from GISAID on 20 August 2024 to confirm that the region targeted by the assay matches recent clade Ib sequences available at the time (2 from Kenya, 2 from Uganda, and 32 from DRC for Clade Ib). The primers and probe sensitivity and specificity were also tested using National Center for Biotechnology Information (NCBI) BLAST against all sequences in NCBI including all orthopoxviruses. No cross reactivity was identified.

In vitro assay testing

The assay was tested in vitro for sensitivity and specificity against extracted nucleic acids from respiratory viruses and non-target nucleic acids (Appendix Table 1). Nucleic-acids were extracted and purified as described below for the wastewater solids samples and then used neat

as template in droplet digital 1-step RT-PCR assays. Assays were run in a single well using the cycling conditions and post processing using a droplet reader as for the wastewater samples in singleplex. No non-target samples produced positive droplets.

To test the quantitative sensitivity of the assay, we challenged the assay to serial dilutions of a positive control. The positive control was a synthetic nucleic acid gene block purchased from IDT (Coralville, Iowa) with the sequence:

ATTAGAATTTTCTATTTCAACGGGTATAGCAGAATATTTGAAACACGGCACTTCGAAATGGAAAAGACTTCCAAACTTAATCACTCCTAGATATTCAGGCGCATATCCACCCACGTGTCAGATTGTTAAATGTCCACATCCTATATCAAACGGAAAACTTCTAGCGGCTTAAAAGATCATACACTCATACAACGACAATGTAGACTTTAAGTGC.

The positive control was serially diluted and then 1.1 µL of each dilution was spiked into 4.4 µL of wastewater solids nucleic-acid extracts known to be negative for the target (collected before outbreak started using methods described below) to produce a total of 5.5 µL of template. The template was used in the PCR reactions as described below. We ran the PCR with and without the RT step to confirm that the inclusion of the RT step would not bias the results. As our project detects mostly RNA viruses, it is economical to use RT-PCR for all targets if possible. Inclusion of the RT step did not affect the quantification of the target (paired t-test, $p = 0.09$) (Figure S2). The assay detected down to at least 2 copies per µl reaction.

Wastewater sample collection for retrospective study

Wastewater samples were obtained from a wastewater treatment plant (WWTP) located in the San Francisco Bay Area serving ≈750,000 people (SEP). Samples were collected using sterile technique in clean, labeled bottles, and were "grab" samples from the primary clarifier. More details about the site are available elsewhere (*2*). We selected samples from 7 days in August 2022 and 3 days in September - October 2024, the majority of which had quantifiable mpox clade II DNA as measured during a prospective, wastewater monitoring program. These samples were chosen and tested for mpox clade Ib to test the specificity of the assay, given the reasonable assumption of the absence of clade Ib DNA in the samples because there had not been any clade I cases reported in the United States at that time. When originally measured during prospective monitoring, the concentration of clade II mpox in each sample (determined as described in detail by Boehm et al. (*2*)) ranged from non-detect to 19,315 cp/g wastewater solids

(Appendix Figure 1). Samples were processed via the methods outlined below for pre-analytical processing and nucleic-acid extraction. Nucleic-acids were stored at −80°C for one to 3 years and underwent one freeze thaw to use as template in RT-PCR as described below.

Wastewater sample collection for prospective study

The process for wastewater collection has been provided elsewhere (*2*) previously and is summarized here. Samples were collected from 147 WWTPs across 40 states between 11/22/2025 and 1/31/2025 approximately three times per week. A full description of the WWTP locations, as well as the number of samples and the time during which they were collected is provided in Appendix Table 2. WWTP staff provided either "grab" samples from the primary clarifier or 24-hour composite samples from the headworks ("influent") (Appendix Table 2 provides sample type for each WWTP). Samples were then stored at 4°C and shipped to the laboratory on ice and processed to completion within 48-hours of receipt at the laboratory. We do not expect the time between sample collection and analysis to affect target quantification as our work and others (*3–5*) show limited decay of the short length nucleic-acid targets over weeks at 4°C. However, it is impossible to rule out the possibility that there is some target lost during sample transport. A total of 3,292 samples were collected and analyzed for this study.

Pre-analytical processing and nucleic-acid extraction of wastewater samples

The pre-analytical processing and nucleic-acid extraction of the samples has been described previously in detail (*2*) and is summarized here briefly. Solids are isolated from wastewater samples and re-suspended in a buffer at a low enough concentration so as to minimize inhibition, and homogenized. Samples were then subjected to nucleic-acid extraction and purification (Chemagic Viral DNA/RNA 300 Kit H96, PerkinElmer, Shelton, CT), and inhibitor removal (Zymo OneStep PCR Inhibitor Removal Kit, Irvine, CA). Dry weight was determined using another aliquot of solids and drying in an oven. Nucleic-acids from 6 to 10 aliquots of each sample were obtained and each used neat as template in 6 to 10 replicate droplet digital 1-step RT-PCR (dd-RT-PCR) reaction wells to measure mpox clade Ib DNA. The process includes negative and positive extraction and PCR controls, as well as a measure of recovery as determined from spiking exogenous bovine coronavirus (BCoV). Further details are described in a protocol on protocols.io (*6*,*7*) and in Boehm et al. (*2*).

Droplet digital PCR

Concentrations of the mpox clade Ib DNA marker in controls and samples were measured in multiplex droplet digital 1-step RT-PCR (dd-RT-PCR) reactions using an AutoDG Automated Droplet Generator (Bio-Rad, Hercules, CA), Mastercycler Pro (Eppendforf, Enfield, CT) thermocycler, and a QX600 Droplet Reader (Bio-Rad). RT-PCR was used for detection and quantification despite the target being genomic DNA because testing is implemented in multiplex with RNA viruses. The mpox clade Ib assay was run using a probe-mixing multiplexing approach using probes labeled with carboxy-X-rhodamine (ROX) and ATTO590 in multiplex with probe-based assays targeting hepatitis A, influenza B, SARS-CoV-2, RSV, influenza A and human norovirus GII as described in Boehm et al. (*2*) For samples processed on and after 27 January 2025, the probe was labeled with ROX alone.

Digital droplet RT-PCR was performed on 20 µl samples from a 22 µl reaction volume, prepared using 5.5 µl template, mixed with 5.5 µl of One-Step RT-ddPCR Advanced kit for Probes (catalog no. 1863021; Bio-Rad), 2.2 µl reverse transcription (RT), 1.1 µl dithiothreitol (DTT), and primers and probes at a final concentration of 900 nM and 250 nM, respectively. Droplets were generated using the AutoDG Automated Droplet Generator (Bio-Rad).

For the in vitro assay testing, a subset of samples was run without the RT step in singleplex (see above). The same mastermix was used, except that the RT and DTT were omitted from the mastermix and replaced with nuclease free water.

PCR was performed using Mastercycler Pro with the following protocol: reverse transcription at 50 °C for 60 min, enzyme activation at 95 °C for 5 min, 40 cycles with 1 cycle consisting of denaturation at 95 °C for 30 s and annealing and extension at 59 °C for 30 s, enzyme deactivation at 98 °C for 10 min, and then an indefinite hold at 4 °C. The ramp rate for temperature changes was set at 2 °C/s, and the final hold at 4 °C was performed for a minimum of 30 min to allow the droplets to stabilize.

Droplets were analyzed using the QX600 Droplet Reader (Bio-Rad). A well had to have over 10,000 droplets for inclusion in the analysis. Extraction and PCR positive and negative controls were run on each 96-well plate. Results from replicate wells were merged for analysis. Concentrations of the targets in wastewater samples are presented as copies per gram dry weight. For a sample to be scored as a positive, there had to be at least 3 positive droplets. The lowest

measurable concentration is ≈1000 copies/g dry weight (corresponds to three positive droplets). Errors are reported as standard deviations (68% confidence intervals) of the measurements as obtained from QX Manager Software (Bio-Rad, version 2.0).

QA/QC

All the negative controls and positive controls were negative and positive for all analyses in this study. Results for recovery of bovine coronavirus were reported elsewhere and indicate near uniform recovery (*2*).

For the prospective study, a total of 274 negative extraction controls and 274 no template controls (NTCs) were run evenly across 274 plates. The negative extraction controls and NTCs were run in 3 wells and the droplet counts were merged across wells (median total accepted droplet counts across negative extraction controls and NTCs were 39816 and 40202, respectively. There were 0 positive droplets in all 274 NTCs. There were 0 positive droplets in 273 of 274 negative extraction controls, and 1 positive droplet in 1 of the negative extraction controls.

In order for a sample to be scored as positive, it had to have 3 or more positive droplets across the merged replicate wells. Across the 3291 negative wastewater solids samples analyzed prospectively in this project, 3186 samples had 0 positive droplets, 84 samples had 1 positive droplet, and 20 samples had 2 positive droplets. The median total accepted droplet count across these samples was 115,050.

Additional details related to the EMMI guidelines

Across all the prospective samples analyzed in this study (n = 3292), the average (standard deviation) number of partitions (droplets) for the across the 6 to 10 replicate wells was 119891 (40375). The volume of the partitions, as reported by the machine vendor is 0.00085 µL. The mean (standard deviation) of copies per partition for each target was 2.73×10^{-7} (1.69×10^{-6}) for mpox clade Ib gene. An example fluorescent plot from the QX600 (6 color reader), as well as the EMMI checklist is included in the Stanford Digital Repository with the deposited data (https://doi.org/10.25740/vk458br0887).

Comparison between SARS-CoV-2 RNA measured in unstored and stored retrospective samples

The samples used for the retrospective study were collected as part of an ongoing wastewater surveillance program. SARS-CoV-2 N gene concentrations were measured in them

in a prospective manner, and the samples were not stored before the quantification of the N gene. Those measurements were previously published (*2*). We compared those N gene measurements to the ones we obtained using the samples after they had been stored between 1 and 3 years to gain insight into potential nucleic-acid degradation. We found a significantly lower concentration between the N gene concentrations measured before and after storage in the 7 samples from 2022 (Appendix Figure 1 samples 1–7, Wilcoxon signed rank test p = 0.02), but no significant differences for the three samples from 2024 (Appendix Figure 1 samples 8–10, p = 0.25). For 2022 samples, the median ratio of the concentrations obtained after storage to those obtained before storage was 0.58 (interquartile range = 0.57 and 0.73), and for 2024 samples the median ratio was 1.57 (IQR = 1.54 and 1.99). Although this analysis was done using an RNA target and there was a statistically significant decrease in concentrations in the older set of samples, it suggests limited degradation in overall concentration and we suspect the same applies to the DNA. Additional work to better understand degradation of nucleic-acids from wastewater during storage would be useful.

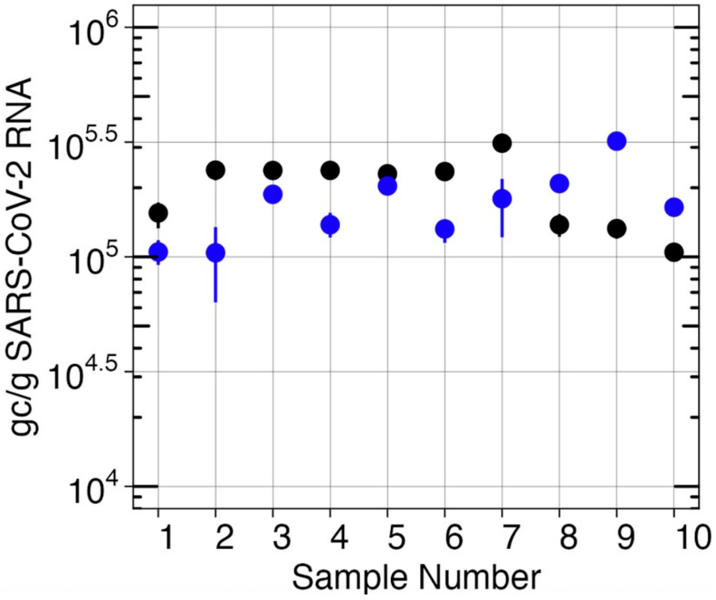

Appendix Figure 1. SARS-CoV-2 concentrations in units of gene copies per gram dry weight solids. Results in black are the concentrations generated when samples were originally tested at the time of collection, as reported by Boehm et al. (*2*). Results generated at the same time as mpox clade Ib testing described in this paper are shown in blue. Error bars that are not visible are smaller than the plotted points. Samples numbers correspond to the following dates, in chronological order: 8/4 - 8/10/2022, 9/3/2024, 9/4/2024, 10/11/2024. Errors represent standard deviations.

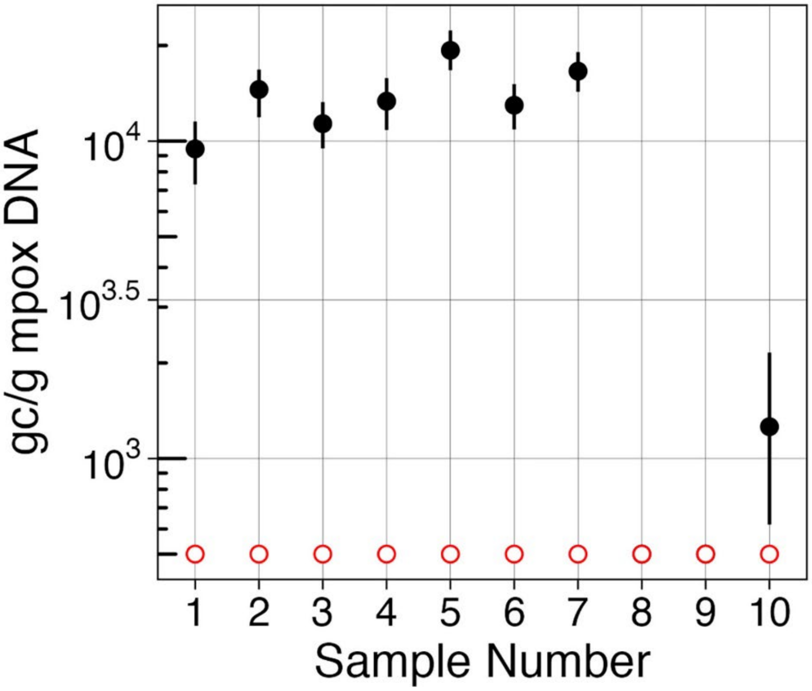

Appendix Figure 2. Mpox clade II concentrations in units of gene copies per gram dry weight solids and mpox clade Ib non-detects. Results for clade II are shown in black and reported by Boehm et al. (*2*). Results for clade Ib are shown in red. Open circles indicate that the target was not detected. Samples numbers correspond to the following dates, in chronological order: 8/4 - 8/10/2022, 9/3/2024, 9/4/2024, 10/11/2024. If a black symbol is not visible, it is also non-detect and under the red symbol. Errors represent standard deviations.

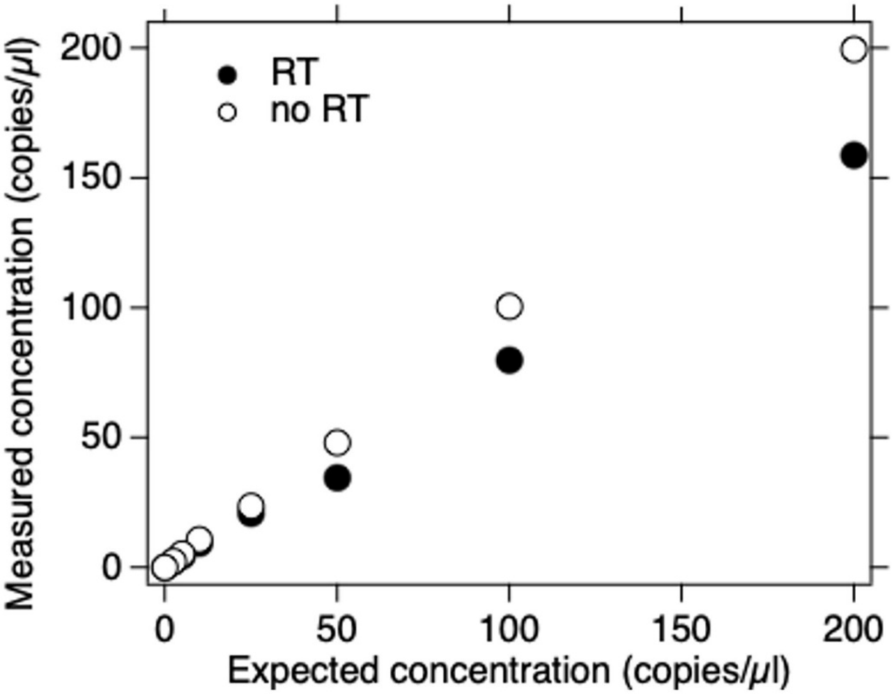

Appendix Figure 3. Concentrations of mpox clade Ib DNA in units of copies per μl of the reaction (total volume is 20 μl). Positive control DNA was spiked into nucleic-acids from wastewater known to be negative for the target and then quantified with and without an RT step (RT and no RT, respectively). Expected concentration is that expected in the sample given what was spiked into it. Measured is the actual measured concentration. Error bars are the standard deviations, but cannot be seen because they are smaller than the symbol.

Appendix Table 1. Pathogens and genetic material used for in vitro specificity testing. Controls from Zeptometrix (Buffalo, NY) are inactivated pathogens. Controls from Twist (South San Francisco, CA) are whole genome synthetic nucleic acid. Controls from ATCC (Manassas, VA) include extracted and synthetic nucleic acids as described in the table.

Zeptometrix	Twist	ATCC
Parainfluenza 1, Parainfluenza 2, Parainfluenza 3, Parainfluenza 4, Influenza A, H1N1pdm, Influenza AH1, Influenza AH3, Influenza B, Adenovirus 1, Adenovirus 3, Adenovirus 31, Rhinovirus Type 1A, RSV A, SARS-CoV-2, M. pneumoniae, C. pneumoniae, Metapneumovirus 8, Coronavirus HKU-1, Coronavirus 229E, Coronavirus NL63, Coronavirus OC43, B. parapertussis, B. pertussis, RSV B	Influenza B, Influenza A H1N1, Influenza A H3N2, Mpox Clade Ia, Mpox Clade II	Genomic SARS-CoV-2 gRNA (extracted), Measles (entire RNA genome, synthetic), Parvovirus B19 (fragments from VP1, VP2, and NS1 regions, synthetic)

Appendix Table 2. Each WWTP included in this study in each row. Solids were obtained the liquid samples by centrifugation as described elsewhere (*2*). The number replicates indicates whether there are 6 or 10 replicates run as described elsewhere (*2*).

State	Plant	Site Name	County	Population	First Sample Date	Last Sample Date	Sample Count	Sample Type	Number of replicates
AK	Anchorage, AK	John M. Asplund Water Pollution Control Facility	Anchorage	220,000	12/17/24	1/30/25	19	Liquids	6
AL	Bessemer, AL	Valley Creek Water Reclamation Facility	Jefferson	225,000	12/13/24	1/31/25	21	Liquids	6
AL	Cahaba River, Birmingham, AL	Cahaba River Water Reclamation Facility	Jefferson	95,000	12/13/24	1/31/25	23	Liquids	6
AL	Fultondale, AL	Five Mile Creek Water Reclamation Facility	Jefferson	77,000	12/13/24	1/31/25	21	Liquids	6
AL	Pinson, AL	Turkey Creek Water Reclamation Facility	Jefferson	30,000	12/13/24	1/31/25	22	Liquids	6
AL	Village Creek, Birmingham, AL	Village Creek Water Reclamation Facility	Jefferson	200,000	12/13/24	1/31/25	21	Liquids	6
AR	Harrison, AR	City of Harrison Wastewater Treatment Plant	Boone	15,000	12/13/24	1/31/25	22	Liquids	6
CA	Davis, CA	City of Davis Wastewater Treatment Plant	Yolo	68,000	12/13/24	1/29/25	21	Solids	6
CA	Fremont, CA	[Fremont Basin] - Raymond A. Boege Alvarado WWTP	Alameda	229,476	12/12/24	1/30/25	13	Liquids	6
CA	Gilroy, CA	South County Regional Wastewater Authority	Santa Clara	110,338	11/23/24	1/31/25	70	Solids	10
CA	Indio, CA	Valley Sanitary District	Riverside	91,765	12/13/24	1/31/25	22	Solids	6
CA	Lompoc, CA	Lompoc Regional Wastewater Reclamation Plant	Santa Barbara	69,290	12/13/24	1/31/25	22	Liquids	6
CA	Los Angeles County, CA	Joint Water Pollution Control Plant	Los Angeles	3,500,000	12/15/24	1/29/25	21	Liquids	6
CA	Los Angeles, CA	Hyperion Water Reclamation Plant (HWRP)	Los Angeles	4,000,000	12/15/24	1/29/25	21	Liquids	6
CA	Marina, CA	Monterey One Water - Regional Treatment Plant	Monterey	262,000	12/13/24	1/31/25	20	Liquids	6
CA	Merced, CA	Merced Wastewater Treatment Plant	Merced	91,000	12/13/24	1/31/25	22	Solids	6
CA	Napa, CA	Soscol Water Recycling Facility	Napa	83,300	12/13/24	1/31/25	22	Solids	6
CA	Novato, CA	Novato Sanitary District	Marin	53,000	12/13/24	1/31/25	20	Liquids	6
CA	Oceanside San Francisco, CA	Oceanside Water Pollution Control Plant	San Francisco	250,000	11/23/24	1/31/25	69	Solids	10
CA	Ontario, CA	Regional Water Recycling Plant No.1 (RP-1)	San Bernardino	890,000	12/16/24	1/30/25	19	Solids	6

State	Plant	Site Name	County	Population	First Sample Date	Last Sample Date	Sample Count	Sample Type	Number of replicates
CA	Palo Alto, CA	Palo Alto Regional Water Quality Control Plant	Santa Clara	236,000	11/22/24	1/31/25	71	Solids	10
CA	Paso Robles, CA	City of Paso Robles Wastewater Treatment Plant	San Luis Obispo	31,037	12/16/24	1/30/25	20	Solids	6
CA	Redwood City, CA	Silicon Valley Clean Water	San Mateo	199,000	11/25/24	1/31/25	50	Solids	10
CA	Riverside, CA	Riverside Water Quality Control Plant	Riverside	350,000	12/13/24	1/31/25	22	Liquids	6
CA	Sacramento, CA	Sacramento Regional Wastewater Treatment Plant	Sacramento	1,480,000	11/23/24	1/31/25	70	Solids	10
CA	San Diego, CA	E.W. Blom Point Loma Wastewater Treatment Plant	San Diego	2,200,000	12/15/24	1/29/25	16	Liquids	6
CA	San Jose, CA	San Jose-Santa Clara Regional Wastewater Facility	Santa Clara	1,500,000	11/22/24	1/31/25	71	Solids	10
CA	San Rafael, CA	Central Marin Sanitation Agency	Marin	104,250	12/15/24	1/28/25	11	Liquids	6
CA	Santa Cruz, CA	City of Santa Cruz WTF - City Influent	Santa Cruz	160,000	12/12/24	1/30/25	22	Solids	6
CA	Santa Rosa, CA	City of Santa Rosa, Laguna Treatment Plant	Sonoma	230,000	12/15/24	1/29/25	21	Solids	6
CA	Southeast San Francisco, CA	Southeast San Francisco	San Francisco	750,000	11/23/24	1/31/25	66	Solids	10
CA	Sunnyvale, CA	City of Sunnyvale Water Pollution Control Plant	Santa Clara	153,000	11/23/24	1/31/25	69	Solids	10
CA	Turlock, CA	Turlock Regional Water Quality Control Facility	Stanislaus	86,000	12/13/24	1/31/25	22	Liquids	6
CA	Vallejo, CA	Vallejo Flood and Wastewater District Wastewater Treatment Plant	Solano	121,000	12/17/24	1/31/25	21	Liquids	6
CA	Woodland, CA	Woodland Water Pollution Control Facility	Yolo	59,000	12/11/24	1/31/25	17	Liquids	6
CO	North, Parker, CO	Parker Water and Sanitation District North Water Reclamation Facility	Douglas	35,000	12/17/24	1/30/25	16	Liquids	6
CO	South, Parker, CO	Parker Water and Sanitation District South Water Reclamation Facility	Douglas	25,000	12/17/24	1/30/25	16	Liquids	6
CT	Stamford, CT	City of Stamford, Water Pollution Control Authority	Fairfield	140,000	12/13/24	1/31/25	21	Solids	6
DE	Seaford, DE	Seaford Wastewater Treatment Facility	Sussex	13,172	12/13/24	1/31/25	22	Solids	6
FL	Eastern, Orange County, FL	Eastern Water Reclamation Facility	Orange	195,299	12/12/24	1/30/25	16	Solids	6

State	Plant	Site Name	County	Population	First Sample Date	Last Sample Date	Sample Count	Sample Type	Number of replicates
FL	Jupiter, FL	Loxahatchee River Environmental Control District	Palm Beach	90,000	12/16/24	1/31/25	21	Liquids	6
FL	Key Biscayne, FL	MDWASD Central District WWTP	Miami-Dade	829,725	12/15/24	1/28/25	21	Liquids	6
FL	North Miami, FL	MDWASD North District WWTF	Miami-Dade	776,150	12/13/24	1/31/25	22	Liquids	6
FL	Northeast, Saint Petersburg, FL	Northeast Water Reclamation Facility	Pinellas	89,847	12/13/24	1/31/25	19	Liquids	6
FL	Northwest, Orange County, FL	Northwest Water Reclamation Facility	Orange	66,690	12/12/24	1/30/25	16	Solids	6
FL	Northwest, Saint Petersburg, FL	Northwest Water Reclamation Facility	Pinellas	94,218	12/13/24	1/31/25	19	Liquids	6
FL	South, Orange County, FL	South Water Reclamation Facility	Orange	183,009	12/12/24	1/30/25	16	Solids	6
FL	Southwest, Orange County, FL	Hamlin Water Reclamation Facility	Orange	50,000	12/12/24	1/30/25	16	Solids	6
FL	Southwest, Saint Petersburg, FL	Southwest Water Reclamation Facility	Pinellas	47,790	12/13/24	1/31/25	20	Liquids	6
FL	Tallahassee, FL	TPSmith Water Reclamation Facility	Leon	212,065	12/12/24	1/30/25	22	Liquids	6
GA	Big Creek, Roswell, GA	Big Creek Water Reclamation Facility	Fulton	189,593	12/16/24	1/29/25	21	Liquids	6
GA	College Park, GA	Camp Creek Water Reclamation Facility	Fulton	73,821	12/11/24	1/29/25	22	Liquids	6
GA	Columbus, GA	South Columbus Water Resources Facility	Muscogee	278,000	12/16/24	1/29/25	14	Solids	6
GA	Johns Creek, Roswell, GA	Johns Creek Environmental Campus	Fulton	84,486	12/16/24	1/29/25	21	Liquids	6
GA	Little River, Roswell, GA	Little River Water Reclamation Facility	Fulton	12,818	12/16/24	1/29/25	21	Liquids	6
GA	RM Clayton, Atlanta, GA	RM Clayton Water Reclamation Center	Fulton	294,660	12/10/24	1/30/25	20	Liquids	6
GA	South River, Atlanta, GA	South River Water Reclamation Center	Fulton	105,160	12/10/24	1/30/25	20	Liquids	6
GA	Utoy Creek, Atlanta, GA	Utoy Creek Water Reclamation Center	Fulton	70,887	12/10/24	1/30/25	20	Liquids	6
HI	Honouliuli, Honolulu, HI	Honouliuli Wastewater Treatment Plant	Honolulu	300,000	12/16/24	1/29/25	21	Liquids	6
HI	Sand Island, Honolulu, HI	Sand Island Wastewater Treatment Plant	Honolulu	390,000	12/16/24	1/29/25	21	Liquids	6
IA	Clinton, IA	City of Clinton	Clinton	29,300	12/13/24	1/31/25	22	Solids	6
IA	Coralville, IA	Coralville Wastewater Treatment Facility	Johnson	23,000	12/13/24	1/31/25	20	Liquids	6

State	Plant	Site Name	County	Population	First Sample Date	Last Sample Date	Sample Count	Sample Type	Number of replicates
IA	Marshalltown, IA	City of Marshalltown Water Pollution Control Plant	Marshall	27,400	12/12/24	1/30/25	22	Liquids	6
IA	Muscatine, IA	Muscatine STP	Muscatine	24,400	12/12/24	1/30/25	23	Solids	6
IA	Ottumwa, IA	Ottumwa WPCF	Wapello	25,529	12/13/24	1/31/25	22	Liquids	6
ID	Coeur d'Alene, ID	City of Coeur d'Alene Water Resource Recovery Facility	Kootenai	50,540	12/13/24	1/31/25	19	Solids	6
ID	Lander Street, Boise, ID	Lander Street Water Renewal Facility	Ada	108,556	12/13/24	1/31/25	22	Liquids	6
ID	West Boise, ID	West Boise Water Renewal Facility	Ada	186,901	12/13/24	1/31/25	21	Liquids	6
IL	Glen Ellyn, IL	Glenbard Wastewater Authority	DuPage	86,000	12/16/24	1/30/25	19	Solids	6
IL	Wheaton, IL	Wheaton Sanitary District	DuPage	63,000	12/13/24	1/31/25	22	Solids	6
IN	Bloomington, IN	Dillman Road WWTP	Monroe	56,090	12/16/24	1/30/25	16	Liquids	6
IN	Carmel, IN	City of Carmel WWTP	Hamilton	86,000	12/13/24	1/31/25	23	Solids	6
IN	Downtown, Jeffersonville, IN	Jeffersonville Downtown WWTP	Clark	25,000	12/13/24	1/31/25	22	Liquids	6
IN	North, Jeffersonville, IN	North Water Reclamation Facility	Clark	25,000	12/13/24	1/31/25	22	Liquids	6
IN	South Bend, IN	City of South Bend Wastewater Treatment Plant	St. Joseph	130,000	12/12/24	1/30/25	22	Liquids	6
KS	Kaw Point, Kansas City, KS	Municipal Wastewater Treatment Plant No. 1 (Kaw Point)	Wyandotte	90,000	12/10/24	1/30/25	19	Solids	6
KS	Lawrence, KS	Lawrence Kansas River Wastewater Treatment Facility	Douglas	80,000	12/13/24	1/31/25	19	Solids	6
KS	P20, Kansas City, KS	Kansas City Treatment Plant #20	Wyandotte	35,000	12/10/24	1/30/25	19	Solids	6
KS	Salina, KS	Salina Wastewater Treatment Plant	Saline	47,000	12/13/24	1/31/25	22	Solids	6
KS	Wolcott, Kansas City, KS	Wolcott Wastewater Treatment Facility	Wyandotte	15,000	12/10/24	1/30/25	12	Liquids	6
KY	Louisville, KY	Morris Forman Water Quality Treatment Center	Jefferson	423,913	12/17/24	1/29/25	12	Solids	6
LA	East Bank, New Orleans, LA	SWBNO East Bank Wastewater Treatment Plant	Orleans	333,406	12/13/24	1/29/25	18	Liquids	6
LA	West Bank, New Orleans, LA	SWBNO West Bank Wastewater Treatment Plant	Orleans	50,591	12/13/24	1/31/25	21	Liquids	6
MA	Boston, MA	Deer Island Treatment Plant	Suffolk	2,400,000	12/13/24	1/31/25	21	Solids	6
MA	Millbury, MA	Upper Blackstone Clean Water	Worcester	250,000	12/12/24	1/30/25	19	Liquids	6
MD	Hagerstown, MD	Hagerstown Wastewater Treatment Plant	Washington	90,000	12/13/24	1/31/25	22	Liquids	6
MD	Hollywood, MD	Marlay Taylor Water	St. Mary's	55,000	12/13/24	1/29/25	13	Liquids	6

State	Plant	Site Name	County	Population	First Sample Date	Last Sample Date	Sample Count	Sample Type	Number of replicates
		Reclamation Facility							
ME	Bangor, ME	City of Bangor Wastewater Treatment Plant	Penobscot	40,000	12/16/24	1/30/25	18	Solids	6
ME	Lewiston, ME	Lewiston Aburn Water Pollution Control Authority	Androscoggin	60,000	12/13/24	1/30/25	20	Liquids	6
ME	Portland, ME	Portland Water District (East End Wastewater Treatment Facility)	Cumberland	65,000	12/13/24	1/31/25	20	Liquids	6
MI	Ann Arbor, MI	City of Ann Arbor Wastewater Treatment Plant	Washtenaw	125,000	12/13/24	1/31/25	22	Liquids	6
MI	Jackson, MI	Jackson Wastewater Treatment Plant	Jackson	90,000	12/12/24	1/30/25	22	Solids	6
MI	Jenison, MI	Grandville Clean Water Plant	Ottowa	75,000	12/13/24	1/31/25	22	Solids	6
MI	Mt. Pleasant, MI	Mt. Pleasant WRRF	Isabella	21,690	12/13/24	1/29/25	20	Liquids	6
MI	Traverse City, MI	Traverse City Regional Waste Water Treatment Plant	Grand Traverse	30,623	12/16/24	1/30/25	20	Liquids	6
MI	Warren, MI	City of Warren Wastewater Treatment Plant	Macomb	140,000	12/16/24	1/30/25	19	Liquids	6
MN	Mankato, MN	City of Mankato Water Resource Recovery Facility (WRRF)	Blue Earth	70,000	12/16/24	1/31/25	21	Solids	6
MN	Red Wing, MN	Red Wing Wastewater Treatment Facility	Goodhue	16,000	12/13/24	1/31/25	22	Solids	6
MN	Rochester, MN	City Of Rochester MN Water Reclamation Plant	Olmstead	120,000	12/13/24	1/31/25	22	Solids	6
MN	St. Cloud, MN	St. Cloud Nutrient, Energy and Water Recovery Facility	Stearns	120,000	12/13/24	1/31/25	22	Liquids	6
MS	Gautier, MS	2C-Gautier POTW	Jackson	19,008	12/15/24	1/28/25	21	Liquids	6
MS	Pascagoula Moss Point, MS	7 C- Pascagoula Moss Point POTW	Jackson	34,333	12/8/24	1/30/25	24	Liquids	6
NC	Kinston, NC	Johnnie Mosley Regional Water Reclamation Facility	Lenoir	25,000	12/13/24	1/31/25	19	Liquids	6
NC	Wilson, NC	City of Wilson - Hominy Creek Water Reclamation Facility	Wilson	50,000	12/13/24	1/31/25	19	Solids	6
NC	Winston-Salem, NC	Archie Elledge WWTP	Forsyth	92,000	12/13/24	1/31/25	16	Liquids	6
NE	Northeast, Lincoln, NE	Northeast Water Resource Recovery Facility	Lancaster	60,000	12/13/24	1/31/25	22	Liquids	6
NE	Theresa Street, Lincoln, NE	Theresa Street Water Resource Recovery Facility	Lancaster	240,000	12/13/24	1/31/25	22	Liquids	6

State	Plant	Site Name	County	Population	First Sample Date	Last Sample Date	Sample Count	Sample Type	Number of replicates
NH	Dover, NH	City of Dover Wastewater Treatment Facility	Strafford	30,000	12/16/24	1/30/25	20	Liquids	6
NJ	Belmar, NJ	South Monmouth Regional Sewerage Authority	Monmouth	52,672	12/13/24	1/31/25	22	Solids	6
NJ	Bridgeton, NJ	Cumberland County Utilities Authority	Cumberland	50,000	12/13/24	1/31/25	19	Liquids	6
NJ	Bridgewater, NJ	The Somerset Raritan Valley Sewerage Authority	Somerset	130,000	12/13/24	1/31/25	14	Liquids	6
NJ	Newark, NJ	Passaic Valley Sewerage Commission	Essex	1,500,000	12/23/24	1/29/25	13	Solids	6
NJ	Oakhurst, NJ	Township of Ocean Sewerage Authority	Monmouth	50,000	12/13/24	1/31/25	21	Liquids	6
NJ	Union Beach, NJ	Bayshore Regional Sewerage Authority	Monmouth	100,000	12/13/24	1/31/25	22	Solids	6
NV	Las Vegas, NV	Clark County Water Reclamation District (CCWRD) Flamingo Water Resource Center (FWRC)	Clark	990,000	12/13/24	1/31/25	22	Liquids	6
NY	Ithaca, NY	Ithaca Area Wastewater Treatment Facility	Tompkins	90,000	12/16/24	1/29/25	12	Liquids	6
NY	Oswego, NY	City of Oswego Wastewater Treatment Plant	Oswego	30,000	12/13/24	1/31/25	21	Liquids	6
OH	Akron, OH	Akron Water Reclamation Facility	Summit	365,000	12/13/24	1/31/25	21	Solids	6
OH	Youngstown, OH	City of Youngstown Wastewater Treatment Plant	Mahoning	174,000	12/13/24	1/30/25	22	Solids	6
PA	Chester, PA	DELCORA Western Regional Treatment Plant	Delaware	220,000	12/12/24	1/30/25	19	Liquids	6
PA	Harrisburg, PA	Capital Region Water AWTF	Dauphin	125,000	12/13/24	1/31/25	22	Liquids	6
SD	Yankton, SD	City of Yankton Wastewater Treatment Facility	Yankton	20,000	12/12/24	1/30/25	22	Liquids	6
TN	Chattanooga, TN	Moccasin Bend WWTP	Hamilton	400,000	12/11/24	1/31/25	19	Liquids	6
TN	Memphis, TN	M.C. Stiles Wastewater Treatment Facility	Shelby	300,000	12/12/24	1/30/25	22	Liquids	6
TX	Dallas Central, Dallas, TX	DCWT Dallas	Dallas	270,000	12/13/24	1/30/25	20	Liquids	6
TX	Gainesville, TX	City of Gainesville Wastewater Treatment Plant	Cooke	17,300	12/13/24	1/31/25	22	Liquids	6
TX	Garland, TX	City of Garland Rowlett Creek WWTP	Dallas	200,000	12/13/24	1/31/25	19	Solids	6

State	Plant	Site Name	County	Population	First Sample Date	Last Sample Date	Sample Count	Sample Type	Number of replicates
TX	Hollywood Road, Amarillo, TX	Hollywood Road WWTP	Potter	60,000	12/15/24	1/28/25	21	Liquids	6
TX	River Road, Amarillo, TX	River Road WWTP	Potter	140,000	12/15/24	1/28/25	21	Liquids	6
TX	South, Laredo, TX	South Laredo WWTP	Webb	120,000	12/13/24	1/31/25	19	Liquids	6
TX	Southside, Dallas, TX	Southside Wastewater Treatment Plant (City of Dallas)	Dallas	421,700	12/12/24	1/30/25	17	Liquids	6
TX	Sunnyvale, TX	Duck Creek Wastewater Treatment Plant	Dallas	186,000	12/12/24	1/30/25	22	Solids	6
TX	White Rock Central, Dallas, TX	DCWT White Rock	Hunt	630,000	12/13/24	1/30/25	21	Liquids	6
TX	Wichita Falls, TX	Wichita Falls Resource Recovery Facility	Wichita	90,000	12/13/24	1/31/25	22	Solids	6
TX	Woodlands SJRA WWTF No. 1, TX	SJRA WWTF No.1	San Jacinto	65,000	12/13/24	1/31/25	22	Liquids	6
TX	Woodlands SJRA WWTF No. 2, TX	SJRA WWTF No.2	San Jacinto	70,000	12/13/24	1/31/25	22	Liquids	6
TX	Zacate Creek, Laredo, TX	Zacate Creek WWTP	Webb	140,000	12/13/24	1/31/25	19	Liquids	6
UT	Central Salt Lake Valley, UT	Central Valley Water Reclamation Facility	Salt Lake	600,000	12/13/24	1/31/25	22	Solids	6
UT	Provo, UT	Provo City Water Reclamation Facility	Utah	115,000	12/17/24	1/29/25	14	Solids	6
VA	Aquia, Stafford, VA	Aquia Wastewater Treatment Facility	Stafford	100,000	12/16/24	1/31/25	13	Solids	6
VA	Little Falls Run, Stafford, VA	Little Falls Run Wastewater Treatment Facility	Stafford	50,000	12/16/24	1/31/25	13	Solids	6
VT	Essex Junction, VT	City of Essex Junction Wastewater Treatment Facility	Chittenden	30,000	12/13/24	1/31/25	22	Solids	6
VT	Montpelier, VT	Montpelier Water Resource Recovery Facility	Washington	10,100	12/16/24	1/30/25	19	Solids	6
VT	South Burlington, VT	South Burlington-Airport Parkway WWTF	Chittenden	16,000	12/17/24	1/30/25	11	Liquids	6
WA	Snohomish, WA	City of Snohomish Wastewater Treatment Plant	Snohomish	10,150	12/13/24	1/31/25	22	Liquids	6
WI	Wausau, WI	Wausau Waterworks Wastewater Treatment Facility	Marathon	44,000	12/11/24	1/31/25	23	Solids	6
WV	Wheeling, WV	City of Wheeling, Water Pollution Control Division	Ohio	100,000	12/13/24	1/31/25	22	Liquids	6

References

1. Schuele L, Masirika LM, Udahemuka JC, Siangoli FB, Mbiribindi JB, Ndishimye P, et al.; GREATLIFE MPOX group; Collaborators. Real-time PCR assay to detect the novel Clade Ib monkeypox virus, September 2023 to May 2024. Euro Surveill. 2024;29:2400486. PubMed https://doi.org/10.2807/1560-7917.ES.2024.29.32.2400486

2. Boehm AB, Wolfe MK, Bidwell AL, Zulli A, Chan-Herur V, White BJ, et al. Human pathogen nucleic acids in wastewater solids from 191 wastewater treatment plants in the United States. Sci Data. 2024;11:1141. PubMed https://doi.org/10.1038/s41597-024-03969-8

3. Roldan-Hernandez L, Graham KE, Duong D, Boehm AB. Persistence of endogenous SARS-CoV-2 and Pepper Mild Mottle virus RNA in wastewater-settled solids. ACS ES T Water. 2022;2:1944–52. PubMed https://doi.org/10.1021/acsestwater.2c00003

4. Zhang M, Roldan-Hernandez L, Boehm A. Persistence of human respiratory viral RNA in wastewater-settled solids. Appl Environ Microbiol. 2024;90:e0227223. PubMed https://doi.org/10.1128/aem.02272-23

5. Muirhead A, Zhu K, Brown J, Basu M, Brinton MA, Costa F, et al. Zika virus RNA persistence in sewage. Environ Sci Technol Lett. 2020;7:659–64. PubMed https://doi.org/10.1021/acs.estlett.0c00535

6. Topol A, Wolfe M, White B, Wigginton K, Boehm AB. High Throughput pre-analytical processing of wastewater settled solids for SARS-CoV-2 RNA analyses. [cited 2022 Jul 25]. https://www.protocols.io/view/high-throughput-pre-analytical-processing-of-waste-b2kmqcu6

7. Topol A, Wolfe M, Wigginton K, White B, Boehm A. High throughput RNA extraction and PCR inhibitor removal of settled solids for wastewater surveillance of SARS-CoV-2 RNA V.2. [cited 2022 Jul 25]. https://www.protocols.io/view/high-throughput-rna-extraction-and-pcr-inhibitor-r-b2mkqc4w
